# Electroresponsive Silk-Based Biohybrid Composites for Electrochemically Controlled Growth Factor Delivery

**DOI:** 10.3390/pharmaceutics12080742

**Published:** 2020-08-07

**Authors:** Adrián Magaz, Mark D. Ashton, Rania M. Hathout, Xu Li, John G. Hardy, Jonny J. Blaker

**Affiliations:** 1 Department of Materials and Henry Royce Institute, The University of Manchester, Manchester M13 9PL, UK; adrian.magaz@postgrad.manchester.ac.uk; 2 Institute of Materials Research and Engineering (IMRE), Agency for Science, Technology and Research (A*STAR), Singapore 138634, Singapore; 3 Department of Chemistry, Lancaster University, Lancaster LA1 4YB, UK; m.ashton2@lancaster.ac.uk; 4 Department of Pharmaceutics and Industrial Pharmacy, Faculty of Pharmacy, Ain Shams University, Cairo 11566, Egypt; rania.hathout@pharma.asu.edu.eg; 5 Department of Chemistry, National University of Singapore, Singapore 117543, Singapore; 6 Materials Science Institute, Lancaster University, Lancaster LA1 4YB, UK; 7 Department of Biomaterials, Institute of Clinical Dentistry, University of Oslo, 0317 Oslo, Norway

**Keywords:** growth factor, stimuli-responsive delivery, nerve repair, conductivity, biohybrid, silk, reduced graphene oxide

## Abstract

Stimuli-responsive materials are very attractive candidates for on-demand drug delivery applications. Precise control over therapeutic agents in a local area is particularly enticing to regulate the biological repair process and promote tissue regeneration. Macromolecular therapeutics are difficult to embed for delivery, and achieving controlled release over long-term periods, which is required for tissue repair and regeneration, is challenging. Biohybrid composites incorporating natural biopolymers and electroconductive/active moieties are emerging as functional materials to be used as coatings, implants or scaffolds in regenerative medicine. Here, we report the development of electroresponsive biohybrid composites based on *Bombyx mori* silkworm fibroin and reduced graphene oxide that are electrostatically loaded with a high-molecular-weight therapeutic (i.e., 26 kDa nerve growth factor-β (NGF-β)). NGF-β-loaded composite films were shown to control the release of the drug over a 10-day period in a pulsatile fashion upon the on/off application of an electrical stimulus. The results shown here pave the way for personalized and biologically responsive scaffolds, coatings and implantable devices to be used in neural tissue engineering applications, and could be translated to other electrically sensitive tissues as well.

## 1. Introduction

Drug delivery technologies are a multibillion-dollar global industry [1]. Driving the increase in research and development efforts [2] is the market need for devices and ‘smart’ implants or scaffolds capable of delivering active agents at specific rates. These systems can be controlled by either physical (e.g., electromagnetic fields, electrical stimulation, temperature) or (bio)chemical (e.g., enzymes, ions, pH) stimuli, in single or combined mechanisms. These technologies enable greater control over the delivery of drugs compared to traditional systems that rely on passive delivery, which cannot be modified in response to therapeutic demand [3].

Electrical stimulation in particular can offer control over drug delivery according to the strength, duration and frequency of the applied field. Consequently, electroconductive/active biomaterials have received great attention in recent years for wound healing and tissue engineering applications due to their potential to allow direct delivery of electrical signals, which are stimulatory to cells/tissues and further trigger a controlled/responsive release of therapeutics to the site of interest [4], potentially wirelessly [5]. Responsiveness to an electric field is an inherent feature of electroconductive/active materials, and their properties can be tailored to suit the delivery of various pharmacological agents and biomolecules. Conductive components used for biomaterial design range from conjugated polymers (e.g., polypyrrole (PPy), polyaniline (PANI), poly (3,4-ethylenedioxythiophene) (PEDOT) and their copolymers), to metallic nanoparticles and carbon-based materials (e.g., carbon nanotubes and graphene family materials) [6,7,8,9,10,11]. A mechanism of electrical conduction involves electron mobility along the backbone of the conjugated polymers and ionic groups appended to or complexed with the conjugated polymers and electrolytes in the medium [12]. High versatility and functionality can be achieved with carbon-based materials. Graphene derivatives in particular have shown advantageous properties for several electrically sensitive tissues, such as nerves [13], but their use as standalone materials is challenging. Biohybrid composites allow flexibility in manufacturing. Combining a protein found in nature as the matrix phase with a conductive component to provide additional functionality offers a multifunctional platform for developing coatings, implants or scaffolds for tissue engineering to match a variety of tissues in surgical reconstruction and regeneration [14].

The response achieved by releasing domain-sized macromolecular therapeutics (e.g., high-molecular-weight drugs >20 kDa, such as proteins, genes, growth factors or siRNA) is challenging due to their larger molecular dimensions and difficulties in their absorption or adsorption to the carrier system, which is limited by diffusion and surface area [15,16,17]. Most electroresponsive systems described for on-demand drug delivery make use of low-molecular-weight (LMW) drugs, which can be easily embedded in a scaffold. LMW drugs are good candidates for disease treatment as they easily transverse through organs and tissues, but they also impart non-specificity, increase the incidence of side effects and are rapidly eliminated from the body, therefore requiring frequent dosing [17]. Furthermore, most responsive systems suffer from short release duration (in the range of minutes to days) of the therapeutic [18,19,20], while tissue regeneration and repair often requires long-term drug release [21].

In particular, the lifelong disability related to peripheral nerve injury (PNI) continues to be a common condition occurring in about 3–10% of trauma patients, with an estimated one million surgical reconstruction procedures performed annually between Europe and the US [9,22,23]. Peripheral nerve axons can spontaneously regenerate to a certain extent after injury, but the probability of recovery decreases as the level of injury increases. Current therapeutic procedures are surgical, employing biomaterials to bridge nerve defects with varying degrees of success [24,25,26]. To enhance the probability of successful outcomes, the use of pharmacological agents and biomolecules, such as microRNAs or growth factors to promote nerve regeneration have gained attention over the last few years [27,28,29]. Neurotrophins are one of many examples of these, which regulate extracellular signaling, neuronal survival and differentiation and axonal regeneration [30]. Among the wide range of neurotrophins, nerve growth factor (NGF) plays a critical role in the development and phenotype maintenance of the peripheral nervous system [31], assisting the functional recovery of injured nerves (by contributing to maintaining the synaptic activity of neurons, preventing apoptosis or regulating the functions of other cell types [32]). However, the effective administration of a domain-sized macromolecular therapeutic such as NGF remains an issue.

**Scheme 1:**
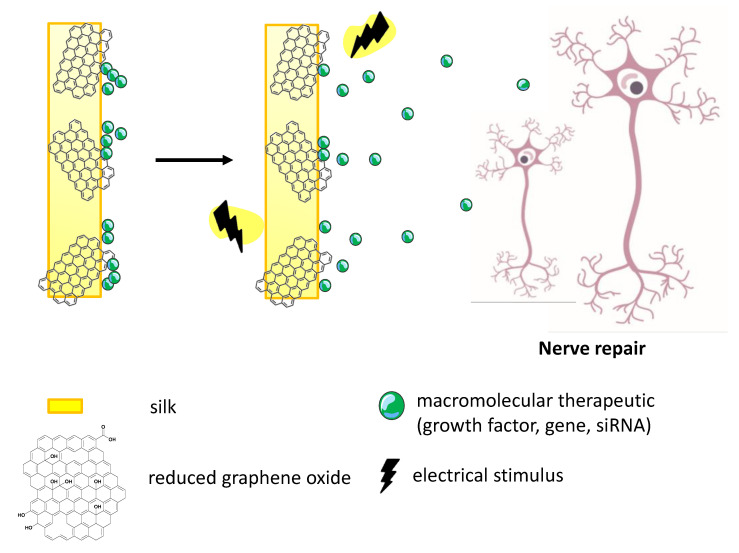
Schematic representation of electroresponsive biohybrid composites based on silk fibroin and reduced graphene oxide for the controlled release of macromolecular therapeutics.

With a view to treat nerve injuries and develop a combinatorial tissue engineered approach, we report here the development of electroresponsive biohybrid composites based on *Bombyx mori* silk fibroin (SF), which acts as the continuous phase, and reduced graphene oxide (rGO) as a conductive dispersed phase. These biohybrid composites enable the controlled release of nerve growth factor-β (NGF-β, 26 kDa) over long-term periods upon the application of a pulsatile electrochemical stimulus (Scheme 1).

## 2. Materials and Methods

### 2.1. Preparation of Regenerated Silk Fibroin

Extraction and purification of silk fibroin (SF) from *Bombyx mori* silkworm cocoons was carried out as previously described [33]. In brief, cocoons (Wildfibres; Birmingham, UK) were dewormed, sliced into small pieces, and degummed in 0.02 M sodium carbonate (Sigma-Aldrich; Gillingham, UK) for 30 min. Degummed SF fibres were thoroughly washed in deionized (DI) water and air-dried overnight. The fibres were then dissolved in 7.9 M lithium bromide (Sigma-Aldrich, UK) solution at 60 °C for 4 h, then dialyzed against 5 L of DI water over the course of 3 days, with regular water changes. The regenerated SF solution was centrifuged, cast onto polystyrene dishes (Sigma-Aldrich, UK) and dried at 60 °C for 2 h in a forced air-circulation oven (Memmert Universal; Schwabach, Germany). The resulting films were subsequently peeled off and kept in sealed vials as stock material for further use.

### 2.2. Preparation of Electroconductive Biohybrid Composite Films

Graphene oxide (GO) flakes (2-DTech; Manchester, UK) (<4 μm lateral width and <2 layers, as quoted by the manufacturer) were dispersed in ≥95% v v^−1^ formic acid (Sigma-Aldrich, UK), sonicated at 80 Hz for 2 h (Elmasonic P60H) (Elma Schmidbauer; Singen, Germany) and homogenized with SF by mixing for 1 h at room temperature. Reduction of GO was performed in situ [34,35] following a previously described protocol [36,37], in which 1 μL of ≥98% v v^−1^ hydrazine monohydrate (Sigma-Aldrich, UK) was added for every 3 mg of GO and heated at 95 °C in an oil bath under constant stirring for a minimum period of 3 h. Film membranes were then prepared by solution casting and evaporation. The prepared silk-based solutions were cast onto polystyrene dishes (Sigma-Aldrich, UK) and the solvent was evaporated overnight in a fume hood, giving films at controlled rGO loadings (control 0, 10, 20, 30 and 40% wt. rGO/SF). The as-prepared films were annealed by immersion in a bath of 80% v v^−1^ ethanol for 20 min to induce β-sheet conformational transition, washed with DI water and dried for 24 h while sandwiched between filter paper (Whatman) (Sigma-Aldrich, UK) to prevent curling or folding. The resulting films were stored in a desiccator until further use.

### 2.3. Characterization of the Electroconductive Biohybrid Composites

The visual appearance of the top surface of the films were observed at room temperature using an optical DS microscope (Olympus) fitted with a digital camera, and the surface was further examined with a JEOL JSM6700F field emission scanning electron microscope (FESEM) (JEOL; Tokyo, Japan). For the latter, samples (*n* = 2 per type) were mounted on aluminum stubs with double-sided adhesive carbon tape and gold-coated prior to visualization at 5 kV, with a working distance of ~8 mm and ×1k magnification.

The chemical structures of the films (*n* = 2 per type) were analyzed with a Fourier transform infrared (FTIR) spectrometer (PerkinElmer 2000) equipped with a zinc selenide (ZnSe) crystal in attenuated total reflectance (ATR) mode. For each measurement, 32 scans were recorded with a resolution of 4 cm^−1^ and wavenumbers from 4000 to 600 cm^−1^.

The swelling characteristics of the films (*n* = 3 per type) were investigated by weight differences between the hydrated and dry states. Samples (15 mm × 15 mm) were immersed in 10 mL of DI water and incubated at 37 °C in a water bath overnight. Excess water was removed by sandwiching the samples in filter paper and weighing immediately afterwards with a high-precision analytical balance. Three measurements were taken per sample. The swelling ratio was calculated according to Equation (1):

(1) $$\mathrm{Swelling}\;\mathrm{ratio}\;\left(\%\right)=\frac{W_{\mathrm{wet}}-W_{\mathrm{dried}}}{W_{\mathrm{dried}}}\times 100$$

Electrical conductivity measurements were performed with a four-point probe automated electrical conductivity and resistivity system (A4P-200 MicroXACT, US) [38], using MicroXACT LabView-based automated software. Conductivity of the samples (*n* = 4 per type) was assessed in different locations across the surface in both the dry and hydrated states. To test the hydrated samples, films were immersed in 10 mL of phosphate buffered saline (PBS) overnight, with excess removed by blotting with tissue paper prior to testing.

### 2.4. In Vitro NGF-β Loading and Release Study

#### 2.4.1. Electrochemical Loading

Recombinant rat nerve growth factor β (NGF-β) (Thermo Fisher Scientific; Altrincham, UK) was diluted into PBS to make a 1 nM solution. The material samples were then actively/electrochemically loaded with NGF-β (using 4 mL of a 1 nM NGF-β solution in PBS as the electrolyte bath). A three-electrode cell was composed of an Ag/AgCl reference electrode, an Au counter electrode and a glassy carbon electrode with the sample material on its surface [1]. Growth factor loading was achieved by applying a constant potential of 0.6 V for 30 min, after which the samples were rinsed in PBS to wash away any residual unincorporated agent.

#### 2.4.2. NGF-β Release 

Chronoamperometric studies were completed using a PalmSens EmStat 3+ potentiostat connected to a computer and PSTrace software (v. 7.4) supplied by Alvatek; Tetbury, UK). The cell comprised a three-electrode system with an Ag/AgCl reference electrode, an Au counter electrode and a glassy carbon working electrode with the sample material on its surface in PBS (0.01 M, 4 mL). Prior to each experiment, there was ‘quiet time’ for 10 s, the initial potential was 0 V, the high potential was 0.7 V, the low potential was −0.5 V, the initial scan was positive, the current was measured at 1 mV intervals, the scan rate used for all experiments was 50 mV s^−1^ and the stimulation lasted 62 s. After stimulation of the material, the cell was allowed to rest for 24 h to allow the released drug to equilibrate in the PBS solution. After allowing the drug to equilibrate in solution post stimulation, a 10 µL aliquot was taken from the electrolyte solution and diluted with 100 µL of PBS before being frozen prior to analysis. Passive release controls were run in parallel with the electrically stimulated samples.

#### 2.4.3. NGF-β Quantification

Quantification of the released NGF-β was achieved by means of ELISA using a commercial kit (Thermo Fisher Scientific, UK). Absorbance measurements were taken in triplicate using a Molecular Devices FlexStation 3 plate reader at 450 nm. Data were reported as the cumulative release by the percentage of the total mass of the growth factor loaded on the films.

### 2.5. In Silico Studies

In silico toxicity screening was carried out using Derek Nexus (v. 6.0.1) (Lhasa Ltd.; Leeds, UK), and the selected structural, topological and physicochemical descriptors were calculated using Bioclipse^®^ (v. 2.6) (Bioclipse project; Uppsala University, Sweden) and Molecular Operating Environment (MOE^®^; v. 2014.0901) (Chemical Computing Group Inc.; Montreal, Canada), with information related to NGF-β sourced from the protein data bank (.pdb) files obtained from the Research Collaboratory for Structural Bioinformatics (RSCB) Protein Data Bank.

### 2.6. Data Analysis

Statistical analysis was performed with GraphPad Prism 8 (San Diego, CA, USA) and checked for normality. Normally distributed data were presented as standard deviation (SD, error bars) of the mean values. For parametric data and multiple comparisons, significance was assessed by one-way ANOVA (one independent variable) or two-way ANOVA (two independent variables), with Tukey’s post hoc analysis test. A value of *p* < 0.05 was considered statistically significant.

## 3. Results and Discussion

**Figure 1:**
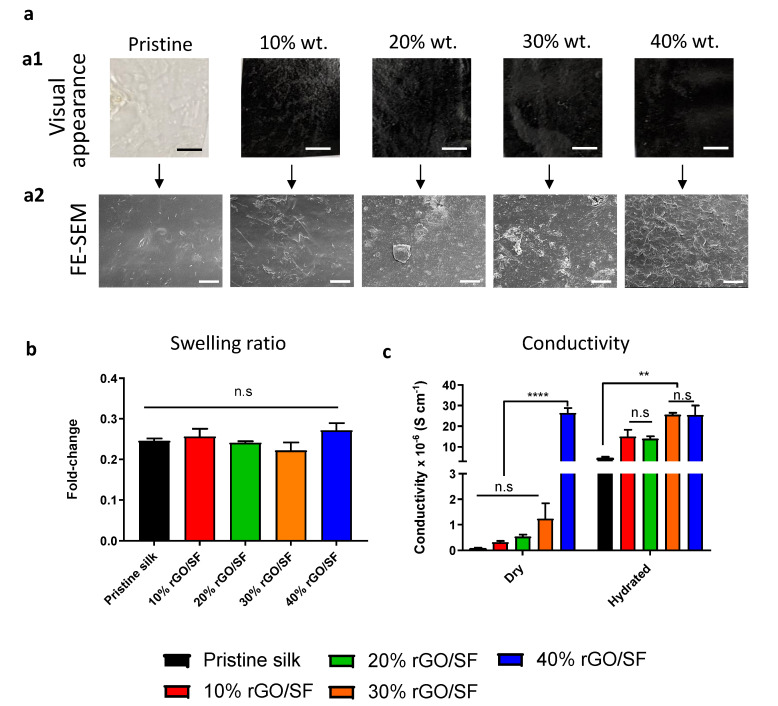
Electroconductive/active silk-based scaffolds. (**a**) Representative (**a1**) macroscopic visual appearance of silk-based films and (**a2**) FESEM micrographs at increasing rGO loading; scale bars at 5 mm and 10 μm respectively. (**b**) Swelling ratio (*n* = 3 per sample type). (**c**) Conductive properties (*n* = 4 per sample type) in the dry and hydrated states. The differences between the experimental groups were analyzed by one-way or two-way ANOVA using Tukey’s post hoc test for multiple comparison. n.s, nonsignificant; ** *p* < 0.01, **** *p* < 0.0001.

Graphene oxide (GO) flakes were homogenized with regenerated silk fibroin in formic acid at high loadings (10–40% wt. GO/SF) and reduced in situ with hydrazine, as detailed in the methodology, until a stable co-suspension was formed. The co-suspensions were cast and the volatiles allowed to evaporate, yielding thin film substrates (thickness < 80 µm) that were annealed in ethanol. The as-cast pristine silk sample was semitransparent to light and became opaque and black after incorporation of rGO (Figure 1(a1)). FE-SEM micrographs of the surface topography of the films (Figure 1(a2)) exhibited differences with rGO incorporation; pristine silk exhibited smooth topography whereas increased topographical features were evident with increased rGO content. *B. mori* silk fibroin-based materials produced from solutions in formic acid tend to be β-sheet rich [39] after evaporation of the formic acid, which renders them insoluble in water. Analysis of the films via attenuated total reflectance infrared spectroscopy (FTIR-ATR) (Figure S1) confirmed the presence of β-sheets in the ethanol-annealed films, exhibiting peaks in the amide I region between 1621 and 1637 cm^−1^ and a peak at 1520 cm^−1^ in the amide II region characteristic of β-sheets [39]. *B. mori* silk fibroin-based materials absorb water, however, we observed no major differences in the swelling ratio of the films with the inclusion of rGO (Figure 1b), with a swelling ratio of ~25% for all films, regardless of rGO content. The mechanism of drug loading and release from a scaffold varies upon the molecular weight of the drug and may be affected by swelling. LMW drugs effectively penetrate the polymer matrix, and their release is more affected by diffusion (e.g., swelling and matrix) and solute size/hydrodynamic radius [40]. A molecular weight cutoff does exist, above which the drug is too large to diffuse into the matrix (e.g., macromolecular therapeutics), so the quantity of the drug loaded is controlled by the surface area available for adsorption [16]. In the scenario of this work, we did not need to consider the influence of swelling on the release of the growth factor since it was electrostatically adsorbed on the surface.

The conductivity of the biohybrid composites was examined in the dry state as well as hydrated state, representing a more physiologically relevant environment (Figure 1c). Pristine silk films showed a conductivity of 9 × 10^−8^ S cm^−1^ in the dry state. The conductivity increased up to 3 × 10^−5^ S cm^−1^ as the content of the filler increased. After hydration, the conductance was dominated by the presence of both the substrate and the electrolytes from the buffered medium, with the conductivity increasing up to three orders of magnitude for the lower rGO loadings (10–30% wt.), analogous to other carbon-loaded materials [41]. The higher conductivity measured in the hydrated state of the samples could be further explained in terms of proton/ion conduction [42,43,44,45] by the *Grotthuss* mechanism, which describes the process of proton hopping across water networks from one water molecule to another across H_9_O_4_^+^ or H_5_O_2_^+^ cations, and could plausibly be enhanced by incorporating pristine graphene [14].

**Figure 2:**
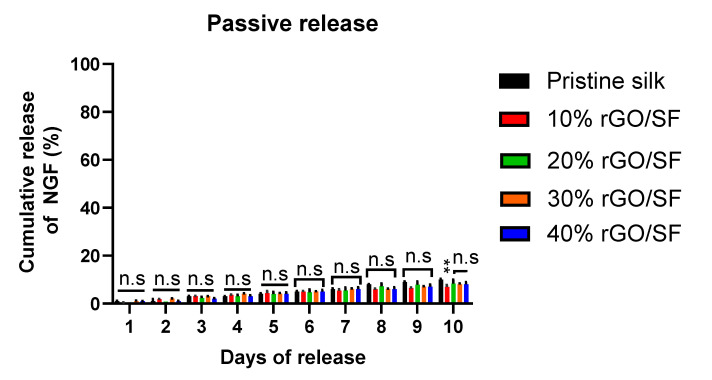
Delivery of nerve growth factor-β (NGF-β) from electroconductive/active silk-based scaffolds. Cumulative release percentage from the films (*n* = 3 per type) with passive release. The differences between the experimental groups were analyzed by two-way ANOVA using Tukey’s post hoc test for multiple comparison (*p*-value with respect to pristine silk). n.s, nonsignificant; ** *p* < 0.01.

**Figure 3:**
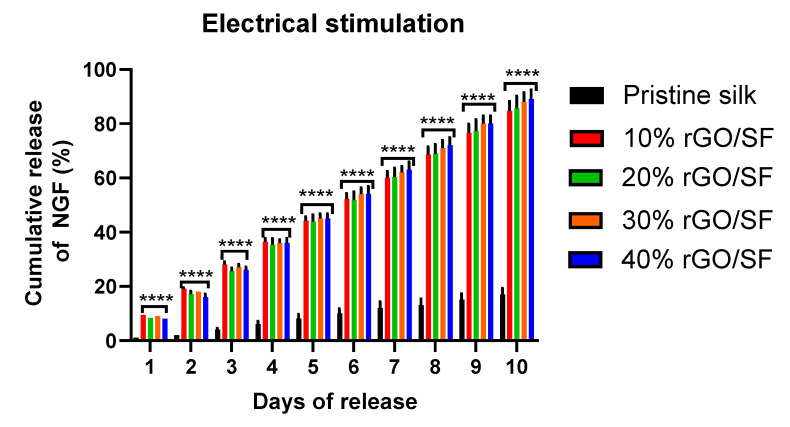
Electrically enhanced delivery of NGF-β from electroconductive/active silk-based scaffolds. Cumulative release percentage from the films (*n* = 3 per type) with electrical stimulation. The differences between the experimental groups were analyzed by two-way ANOVA using Tukey’s post hoc test for multiple comparison (*p*-value with respect to pristine silk). **** *p* < 0.0001.

Myriad applications could be developed using stimuli-responsive drug delivery systems, which can control the chronopharmacology of the therapeutic of interest in line with the chronobiology of the condition to be treated. A variety of different therapeutics have been delivered using electroresponsive conductive scaffolds, as previously reviewed [46,47]. Most of these systems relied on the use of conductive polymers (e.g., PPy, PANI and PEDOT) as the electroconductive/active moiety [46,47]. For instance, electrically controlled drug delivery from GO-nanocomposite PPy and silk-PPy films have been previously reported [18,20]. However, the use of conjugated polymers tends to be limited due to their poor processability in aqueous solution, brittleness and tendency to crack [48]. GO is readily dispersible thanks to oxygen-containing functional groups on its surface, and it stands out for its ease of processability. Graphene-based scaffolds were previously reported as drug delivery systems based on the use of synthetic hydrogels and silk as the host materials [19,49], however, these systems relied on the use of LMW drugs for short-term release. In this work, the prepared film samples were loaded (doped) actively/electrostatically with a high-molecular-weight macromolecule - nerve growth factor-β (NGF-β), and its release profile was determined by means of an enzyme-linked immunosorbent assay (ELISA) which provides increased sensitivity and low limits of detection. The growth factor was released either passively by diffusion (Figure 2, zoomed-in version shown in Figure S2) or actively triggered upon the application of an electrical stimulus (Figure 3, stimulation paradigm depicted in Figure S3). Passive release from the composite films was observed (ca. 1–2% at each time-point tested), with a cumulative release of <10% over the course of the 10-day experiment. The application of a reducing potential to the NGF-β-doped films triggered the release of the growth factor.

Notable differences were observed between the electrically stimulated samples and the non-stimulated ones. In particular, the amount of NGF-β released from the electrically stimulated films was significantly higher (five- to eight-fold increase) than for the non-stimulated films (i.e., passive release) at each time point tested, with an enhancement of up to 8–10% release at each time-point. By day 10, most of the loaded therapeutic had been released from the samples via electrical stimulation. The release of drugs over prolonged time periods is necessary for tissue repair and regeneration [21,50]; it obviates the need for repeated high dosing and improves the therapeutic index [17]. In addition to providing long-term release, the system described in this work could be further modulated in terms of the frequency pulse and duration used for stimulation. Notable differences were also observed for the pristine silk sample, for which electrical stimulation resulted in ca. <20% cumulative drug release over the course of 10 days, in comparison to the almost complete release of the growth factor from the composite films. This clearly demonstrated the benefit of the presence of a conductive carbon-based dispersed phase in the hybrid composites, which enhances the electrically triggered release of this therapeutic. However, no major differences in the drug release profiles were observed for the different rGO loadings, likely because the conductivities of the samples in the hydrated state were similar. The active delivery of NGF-β controlled in an on/off fashion is particularly interesting regarding the potential control of its chronopharmacology for nerve repair. A variety of other therapeutics or biological molecules (e.g., proteins, genes, siRNA molecules) could be delivered in a similar fashion when exposed to electrical fields, where the loading and release profiles of the bioactives could be correlated with their molecular descriptors (Tables S1 and S2).

An important factor in the design of advanced composite biomaterials are material–tissue interactions, particularly the hazards that such materials present to the environment and life [51,52]. In vitro and in vivo tests showed that the choice of the specific graphene-based material incorporated in the composites and the method of use are of key importance regarding the risks associated with the application of such materials for biomedical applications [51,52]. Our in silico toxicity screening studies of these nanomaterials (Table S2) using Derek Nexus (Derek Nexus: 6.0.1, Nexus: 2.2.2) [53] confirmed some potential for graphene, GO and rGO to induce skin sensitization due to the presence of conjugated dienes in their structures, supported by observations in both in vitro and in vivo models [54,55,56,57,58]. Moreover, hydroxynaphthalene derivatives such as rGO were demonstrated as estrogen receptor modulators [59]. Our in silico mutagenicity screening studies of these nanomaterials (Table S2) using Sarah Nexus (Sarah Nexus: 3.0.0, Sarah Model: 2.0) also suggested that graphene, GO and rGO may be mutagenic, supported by in vitro and in vivo studies using graphene quantum dots [60], GO [61] and rGO [62]. Therefore, the specific method to use drug delivery devices based on these composites needs to be considered. In the case of the materials described in this work, it is possible to contemplate their use as microneedle patches or coatings on medical devices that would be removed after use and/or disposed of.

## 4. Conclusions

We report the development of electroresponsive biohybrid composites based on silk fibroin and reduced graphene oxide allowing the controlled release of a high-molecular-weight therapeutic over prolonged periods of time. NGF-β was loaded electrostatically and its release was facilitated over 10 days by electrical stimulation in an on/off mechanism, with a five- to eight-fold increase compared to the use of unmodified silk or the passive diffusion route. The conductivity of the system can be tuned as the ratio of its components changes, and the release profile can be controlled by triggering. These electroresponsive biohybrid composites based on silk could be manufactured in various other forms (e.g., sponges, aligned fibres or guidance conduits) to potentially regenerate nerve tissue. The concept of introducing electroconductive/active moieties into the network to impart conductive properties to protein-based systems could be readily applied to other polypeptides found in nature or recombinantly synthesized. The applications of the described system are numerous, from conductive scaffolds that can be implanted directly into electrically excited tissues other than nerve to in vitro platforms to grow electrically sensitive cells ex vivo. These findings represent a step forward in the generation of biohybrids easily tailorable to multiple biological applications, where different levels of conductivity may be desired.

## Supplementary Materials

The following are available online at https://www.mdpi.com/1999-4923/12/8/742/s1, Figure S1: FTIR-ATR spectrum of the electroconductive/active silk-based films. Figure S2: Zoomed-in cumulative NGF-β release (passive release) from the films. Figure S3: Electrical stimulation paradigm. Table S1: Molecular descriptors. Table S2: Molecular-input line-entry system notations of the nanomaterials and molecules studied.
