# Supplementary material for: Electroresponsive Silk-Based Biohybrid Composites for Electrochemically Controlled Growth Factor Delivery
Source: Pharmaceutics. 2020 Aug 7;12(8):742. doi: 10.3390/pharmaceutics12080742 (PMC7463593; doi:10.3390/pharmaceutics12080742)
Supplement: Supplementary file 1 [file pharmaceutics-12-00742-s001.pdf]

pharmaceutics

MDPI

Supplementary Materials: Electroresponsive Silk-Based Biohybrid Composites for Electrochemically Controlled Growth Factor Delivery

Adrián Magaz, Mark D. Ashton, Rania M. Hathout , Xu Li , John G. Hardy and Jonny J. Blaker

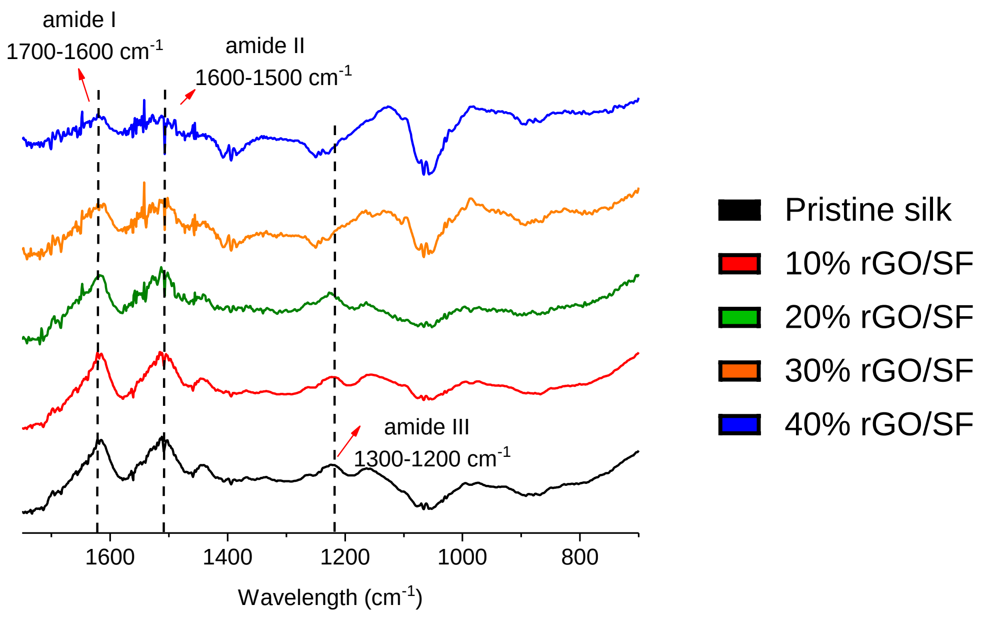

Figure S1. Chemical structure of silk. Representative FTIR-ATR spectrum of the electroconductive/active silk-based films.

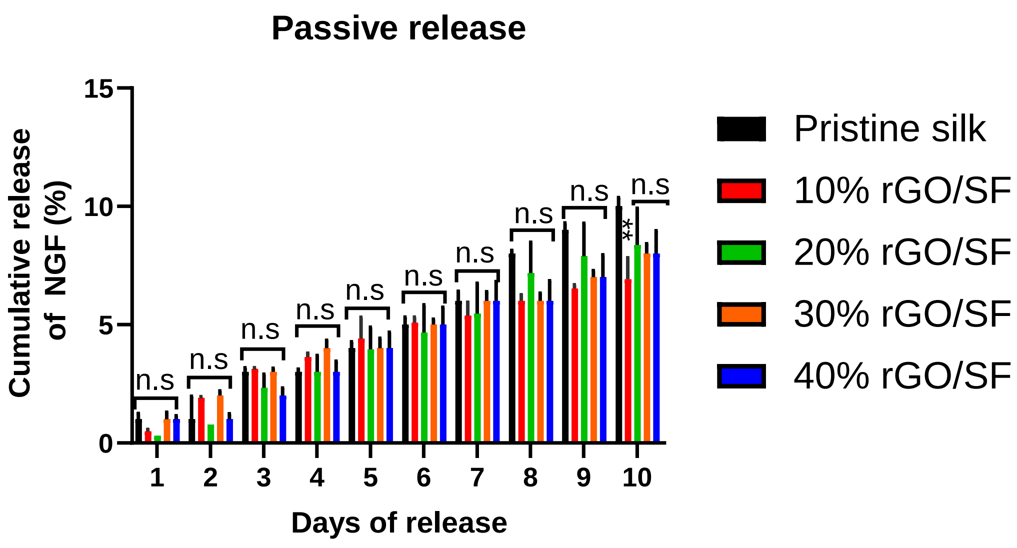

Figure S2. Re-scaled version of Figure 2 of the main manuscript showing cumulative NGF-β release from the films (n=3 per type); passive release. The differences between the experimental groups were analyzed by two-way ANOVA using Tukey's post hoc test for multiple comparison (p-value with respect to neat silk). n.s non-significant; **p<0.01.

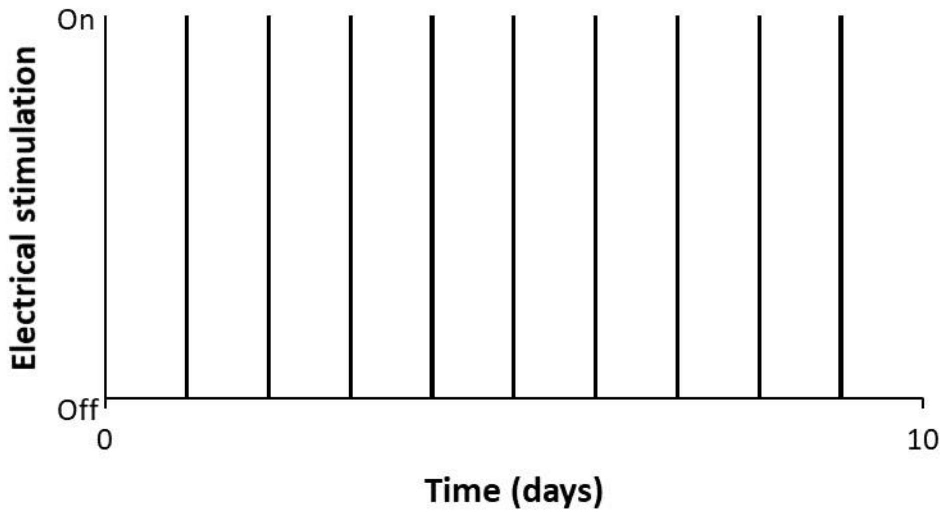

Figure S3. Electrical stimulation paradigm. 10 cycles of: 62 seconds on, 24 hours off.

Table S1. Molecular descriptors.

PDB Code	5LSD
Total Hydrophobic surface area	14006.4
No. of H-bond acceptors	302
No. of H-bond donors	304
Molecular globularity	0.21105774
Molecular flexibility	535.1393
Total aromatic bonds	208
Molecular weight (g mol^{-1})	26518.16

Table S2. Simplified molecular-input line-entry system (SMILES) notations for fragments of the nanomaterials and NGF-β studied.

**Graphene:**
C1=CC=C3C2=C1C=C6C=5C2=C4C(=C3)C=C9C8=C4C=7C=5C(C=C6)=CC=%11C=7C=%10C8=C(C=C9)C=C%12C=%10C(C=%11)=CC=C%12
**Graphene oxide (GO):**
C1=C(C=C3C2=C1C=C6C5(C2=C4C(=C3)C=CCC4C78C5(C(CC6)CCC7)O8)O)O
**Reduced graphene oxide (rGO):**
C1=C(C3=C2C(=C1)C(=C5C4=C2C(C=C3)=CCC4=CC=C5)C(O)=O)O
**NGF-β:**
S1SCC2NC(=O)C(NC(=O)C(NC(=O)C(NC(=O)C(NC(=O)C(NC(=O)C(NC(=O)C(NC(=O)C(NC(=O)C(NC(=O)CNC(=O)C(NC(=O)C3NC(=O)CNC(=O)C(NC(=O)C(NC(=O)C(NC(=O)C4N(CCC4)C(=O)C(NC(=O)CNC(=O)C(NC(=O)C(NC(=O)C(NC(=O)C(NC(=O)C(NC(=O)C(NC(=O)C(NC(=O)C(NC(=O)C(NC(=O)CNC(=O)C(NC(=O)C(NC(=O)C(NC(=O)C(NC(=O)C(NC(=O)C(NC(=O)C(NC(=O)C(NC(=O)C(NC(=O)C(NC(=O)C(NC(=O)CNC(=O)C(NC(=O)C(NC(=O)C(NC(=O)C(NC(=O)C4N(CCC4)C(=O)C(NC(=O)C(NC(=O)C(NC(=O)C([NH3+])CO)CO)C(O)C)Cc4[nH+]c[nH]c4)C(C)C)Cc4ccccc4)Cc4[nH+]c[nH]c4)CCSC)CCC(=O)[O-])Cc4ccccc4)CO)C(C)C)C1)CC(=O)[O-])CO)C(C)C)CO)C(C)C)Cc1c4c([nH]c1)cccc4)C(C)C)CC(=O)[O-])CCCC[NH3+])C(O)C)C(O)C)C)C(O)C)CC(=O)[O-])C(CC)C)CCCC[NH3+])CCCC[NH3+])CCC(=O)[O-])C(C)C)C(O)C)C(C)C)CC(C)C)C)CCC(=O)[O-])C(C)C)CC(=O)N)C(CC)C)CC(=O)N)CC(=O)N)CO)C(C)C)Cc1ccccc1)CCCNC(=[NH2+])N)CCC(=O)N)Cc1ccc(O)cc1)Cc1ccccc1)Cc1ccccc1)CCC(=O)[O-])C(O)C)CCCC[NH3+])CSSCC(NC(=O)C(NC2=O)C(O)C)C(O)C)C(O)C)Cc1[nH+]c[nH]c1)C(O)C)Cc1ccccc1)C(C)C)CCCC[NH3+])C)CC(C)C)C(O)C)C(O)C)CC(=O)[O-])CCC(=O)[O-])CCCC[NH3+])CCC(=O)N)C)C)Cc1c2c([nH]c1)cccc2)CCCNC(=[NH2+])N)Cc1ccccc1)C(CC)C)CCCNC(=[NH2+])N)C(CC)C)CC(=O)[O-])C(O)C)C)C(=O)NC(C(C)C)C(=O)NC(CSSC3)C(=O)NC(C(C)C)C(=O)NC(CC(C)C)C(=O)NC(C(=O)NC(CCCNC(=[NH2+])N)C(=O)NC(CCCC[NH3+])C(=O)NC(C(=O)NC(C(O)C)C(=O)NC(CCCNC(=[NH2+])N)C(=O)[O-])C)CO)CCCNC(=[NH2+])N)C)CO)CC(=O)N)C(C)C)CCC(=O)[O-])CO)CCCNC(=[NH2+])N)C(CC)C)CC(=O)[O-])CO)CCCC[NH3+])Cc1[nH+]c[nH]c1)Cc1c2c([nH]c1)cccc2)CC(=O)N)CO)Cc1ccc(O)cc1.S1SCC2NC(=O)C(NC(=O)C(NC(=O)C(NC(=O)C(NC(=O)C(NC(=O)C(NC(=O)C(NC(=O)C(NC(=O)C(NC(=O)CNC(=O)C(NC(=O)C3NC(=O)CNC(=O)C(NC(=O)C(NC(=O)C(NC(=O)C4N(CCC4)C(=O)C(NC(=O)CNC(=O)C(NC(=O)C(NC(=O)C(NC(=O)C(NC(=O)C(NC(=O)C(NC(=O)C(NC(=O)C(NC(=O)C(NC(=O)CNC(=O)C(NC(=O)C(NC(=O)C(NC(=O)C(NC(=O)C(NC(=O)C(NC(=O)C(NC(=O)C(NC(=O)C(NC(=O)C(NC(=O)C(NC(=O)CNC(=O)C(NC(=O)C(NC(=O)C(NC(=O)C(NC(=O)C4N(CCC4)C(=O)C(NC(=O)C(NC(=O)C(NC(=O)C([NH3+])CO)CO)C(O)C)Cc4[nH+]c[nH]c4)C(C)C)Cc4ccccc4)Cc4[nH+]c[nH]c4)CCSC)CCC(=O)[O-])Cc4ccccc4)CO)C(C)C)C1)CC(=O)[O-])CO)C(C)C)CO)C(C)C)Cc1c4c([nH]c1)cccc4)C(C)C)CC(=O)[O-])CCCC[NH3+])C(O)C)C(O)C)C)C(O)C)CC(=O)[O-])C(CC)C)CCCC[NH3+])CCCC[NH3+])CCC(=O)[O-])C(C)C)C(O)C)C(C)C)CC(C)C)C)CCC(=O)[O-])C(C)C)CC(=O)N)C(CC)C)CC(=O)N)CC(=O)N)CO)C(C)C)Cc1ccccc1)CCCNC(=[NH2+])N)CCC(=O)N)Cc1ccc(O)cc1)Cc1ccccc1)Cc1ccccc1)CCC(=O)[O-])C(O)C)CCCC[NH3+])CSSCC(NC(=O)C(NC2=O)C(O)C)C(O)C)C(O)C)Cc1[nH+]c[nH]c1)C(O)C)Cc1ccccc1)C(C)C)CCCC[NH3+])C)CC(C)C)C(O)C)C(O)C)CC(=O)[O-])CCC(=O)[O-])CCCC[NH3+])CCC(=O)N)C)C)Cc1c2c([nH]c1)cccc2)CCCNC(=[NH2+])N)Cc1ccccc1)C(CC)C)CCCNC(=[NH2+])N)C(CC)C)CC(=O)[O-])C(O)C)C)C(=O)NC(C(C)C)C(=O)NC(CSSC3)C(=O)NC(C(C)C)C(=O)NC(CC(C)C)C(=O)NC(C(=O)NC(CCCNC(=[NH2+])N)C(=O)NC(CCCC[NH3+])C(=O)NC(C(=O)NC(C(O)C)C(=O)NC(CCCNC(=[NH2+])N)C(=O)[O-])C)CO)CCCNC(=[NH2+])N)C)CO)CC(=O)N)C(C)C)CCC(=O)[O-])CO)CCCNC(=[NH2+])N)C(CC)C)CC(=O)[O-])CO)CCCC[NH3+])Cc1[nH+]c[nH]c1)Cc1c2c([nH]c1)cccc2)CC(=O)N)CO)Cc1ccc(O)cc1
